# Efficiency of Hyaluronic Acid in Infrabony Defects: A Systematic Review of Human Clinical Trials

**DOI:** 10.3390/medicina58050580

**Published:** 2022-04-23

**Authors:** Florin Onisor, Simion Bran, Alexandru Mester, Andrada Voina-Tonea

**Affiliations:** 1 Department of Maxillofacial Surgery and Implantology, University of Medicine and Pharmacy “Iuliu Hatieganu”, 400012 Cluj-Napoca, Romania; florin.onisor@umfcluj.ro (F.O.); dr_brans@umfcluj.ro (S.B.); 2 Department of Oral Health, University of Medicine and Pharmacy “Iuliu Hatieganu”, 400012 Cluj-Napoca, Romania; 3 Department of Dental Materials, University of Medicine and Pharmacy “Iuliu Hațieganu”, 400012 Cluj-Napoca, Romania; andrada.tonea@umfcluj.ro

**Keywords:** hyaluronic acid, infrabony defect, open-flap debridement, periodontal surgery

## Abstract

*Background and objectives:* The aim of this systematic review was to assess the electronic literature about the benefits of using hyaluronic acid (HA) in the surgical periodontal treatment of infrabony defects. *Materials and methods:* This review was conducted under the PRISMA guidelines. The electronic search was conducted on PubMed, Scopus, Web of Science, and Cochrane databases until February 2022. The inclusion criteria consisted of human clinical trials that reported the use of HA in open-flap debridement (OFD) for infrabony defects. The assessment of risk of bias was performed using the Cochrane risk of bias tool. Statistical analysis was performed using Review Manager. *Results:* Overall, three RCTs were found eligible for the statistical analysis. Probing depth (PD) reduction and clinical attachment level (CAL) gain in the HA test group presented WMs of −1.11 mm (95% CI −2.38 to 0.16 mm; *p* = 0.09) and −1.38 mm (95% CI −2.26 to −0.49 mm; *p* = 0.002), respectively. However, the heterogeneity of the RCTs was high, and the risk of bias, in general, was low. *Conclusions:* The use of hyaluronic acid seems to have beneficial effects in periodontal surgery using OFD, in terms of PD and CAL. To draw a clear conclusion, more adapted and well-designed clinical trials are needed to assess the advantage of this product in comparison with other products.

## 1. Introduction

Hyaluronic acid (HA) represents one of the main constituents of the connective tissue. HA belongs to the class of glycosaminoglycans, known for containing an elevated molecular mass, which can also be found in other human tissues such as tegument or vitreous fluid [1]. In comparison to the cementum or the alveolar bone, HA is present in a higher concentration in the connective tissue of the periodontal ligament and the gingival tissue. When chronic periodontitis is accompanied by inflammation or an inflammatory–dystrophic process, hyaluronidases produced by microorganisms determine the breakdown of HA. As a result, the use of external HA to repair the periodontium was suggested by Kopchak and coworkers [2].

It is well known that periodontal disease may lead to the formation of bone defects. Periodontal bone defects are classified by osseous resorption tendencies. Defects with a pocket base positioned coronally in relation to the alveolar bone are defined as “suprabony defects”, while “infrabony defects” are considered when an apical position of the pocket base is in relation to the alveolar bone crest [3]. Currently, the treatment of infrabony defects can vary from nonsurgical to surgical therapy [3,4,5]. Currently, various surgical therapies are available for treating infrabony defects, from open-flap procedures, associated with guided tissue regeneration [4], to autologous or synthetic bone grafts [5].

In periodontal disease treatment, HA may play an important role in both surgical and nonsurgical approaches [6,7]. In order to reestablish a healthy periodontium, HA treatment has demonstrated antimicrobial, anti-inflammatory, and anti-welling properties. Furthermore, HA is able to balance the levels of antioxidants and reactive oxygen microorganism species [6]. In some cases, HA can even offer further advantages, related to clinical attachment levels or probing depth improvement [7].

Taking into consideration that a variety of functions are performed by HA, ranging from cell attachment and migration to cellular development [8], the role of HA in the treatment of infrabony defects may be significant. Therefore, the aim of our study was to systematically review the electronic literature about the efficiency of hyaluronic acid in the surgical treatment of infrabony defects.

## 2. Materials and Methods

This systematic review was written under the guidelines of the Preferred Reporting Items for Systematic Review and Meta-Analysis (PRISMA) [9]. Our review question was as follows: What is the efficacy of hyaluronic acid in infrabony defects for periodontal regeneration compared to open-flap debridement alone in human clinical trials?

### 2.1. Eligibility Criteria

The following inclusion criteria were established according to the PICOS framework:

— Participants (P), patients with infrabony defects;
— Intervention (I), open-flap debridement + addition of hyaluronic acid;
— Comparison (C), open-flap debridement alone;
— Outcome (O), regenerative potential of hyaluronic acid in terms of periodontal indices (probing depth, clinical attachment level) with follow-up (12 months);
— Study type (S): split or parallel randomized clinical trials (RCTs), published in the English language.

The exclusion criteria consisted of articles published in a language other than English, reviews, case reports, prospective/retrospective studies, in vivo and in vitro studies, chapters, monographs, and incomplete or unpublished data.

### 2.2. Literature Search

An electronic search on four databases, PubMed (Medline), Scopus, Web of Science, and Cochrane, was assessed to find all relevant RCTs published until February 2022. Furthermore, a gray literature search was conducted on ClinicalTrials.gov (accessed on 21 March 2022), OpenGrey, and the World Health Organization (WHO) International Clinical Trials Registry Platform. A manual search of the potential studies was performed in the following periodontology journals: Journal of Periodontal Research, Journal of Periodontology, Journal of Clinical Periodontology, Clinical Oral Investigations, International Journal of Periodontics and Restorative Dentistry, Journal of Dentistry, and Journal of Indian Society of Periodontology.

The following keyword combination was used: (“hyaluronic acid”) AND (“infrabony defect” OR “infrabony lesion” OR “intrabony defect” OR “intrabony lesion” OR “intraosseous defect” OR “angular defect” OR “periodontal defect” OR “periodontal lesion” OR “open-flap debridement” OR “periodontal debridement” OR “guided tissue regeneration” OR “periodontal surgery”). The first step consisted of the identification of titles and abstracts considered eligible and the exclusion of irrelevant articles. In the second step, full-text papers previously shortlisted were read, analyzed, and considered according to the inclusion criteria. In the third step, a final decision was made in order to include RCTs relevant to our subject.

### 2.3. Data Extraction

The data from the RCTs included were extracted following a standardized data form: first author, year of study, country, type of study, patients’ characteristics, periodontal indices, type of intervention, results, and conclusions.

### 2.4. Risk of Bias

For each RCT included, risk of bias was calculated using the risk of bias tool (version 2.0) from Cochrane Collaboration [10]. The quantification was applied for the following domains: random sequence generation, allocation concealment, blinding of outcome assessment, incomplete outcome data, selective reporting, and other bias. The domains were judged by two independent reviewers and were evaluated as high, unclear, or low. If any disagreement was present, a third reviewer intervened to resolve the issue.

### 2.5. Statistical Analysis

In this review, PD (from the gingival margin to the base of the pocket) and CAL (from CEJ to the base of the pocket) parameters were quantified with a follow-up of 12 months. The 95% confidence interval (CI) and weighted means (WMs) were calculated using a random-effects model for continuous data between test and control group. Results were presented using forest plots graphics. The heterogeneity of the RCTs was assessed using the I^2^ statistic, where I^2^ > 50% represented high heterogeneity. Statistical analysis was conducted using Review Manager 5.4.1 version. Statistical significance was considered for *p* < 0.05.

## 3. Results

### 3.1. Search Results

**Figure 1:**
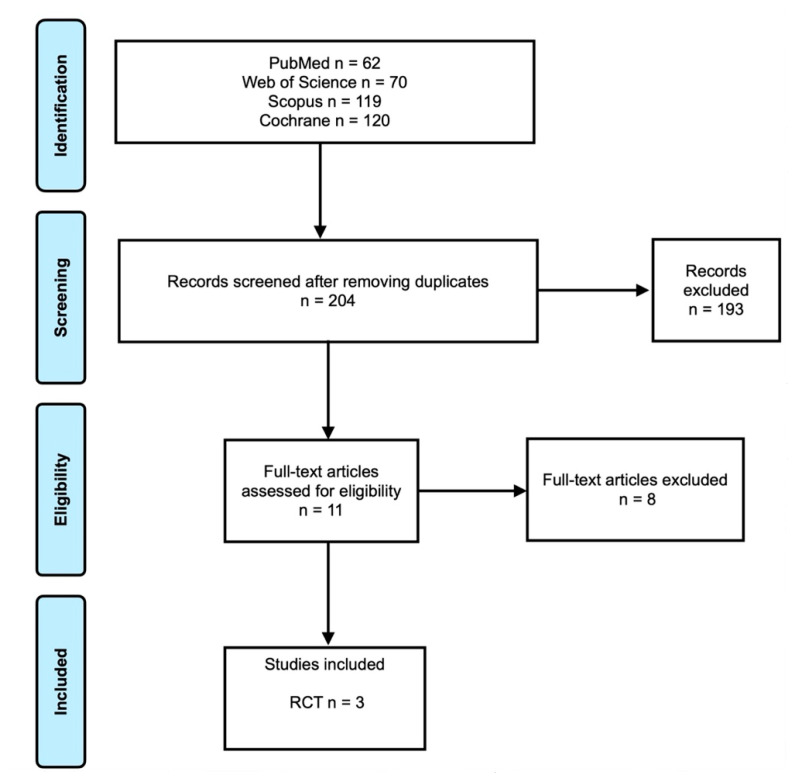
Prisma flowchart.

The electronic search yielded 62 papers in PubMed, 70 papers in Web of Science, 119 papers in Scopus, and 120 papers in Cochrane database (Figure 1). After removing duplicates, 204 papers were assessed in terms of titles and abstract. For full-text assessment, 11 papers were considered. In the end, three RCTs were considered. Reasons for the exclusion of articles can be found in Table 1. No relevant results were found from the gray literature and manual search.

### 3.2. General Characteristics

The three RCTs [19,20,21] included were published between 2013 and 2021 (Table 2). Regarding the type of study, one was a parallel RCT [19], while two were split-mouth RCTs [20,21]. The studies were conducted in Italy, Brazil, and India. The sample size varied from 20 patients to 40 patients. All RCTs reported the age and the sex of the participants.

### 3.3. Treatment of Infrabony Defects for Test and Control Groups

Briguglio and coworkers [19] conducted a parallel RCT. Each patient received SRP therapy and oral hygiene instructions. After maintenance therapy, patients with infrabony defects with a PD interproximal ≥ 5 mm were considered for surgery. The test group was treated with the OFD procedure plus HA, while the control group was treated with only OFD. All flaps were closed with interrupted sutures. The authors did not mention the dosages used for HA application. At the end of the procedure, each patient received home oral hygiene instructions.

Santana and coworkers [20] only considered infrabony defects with a PD higher than 6 mm as eligible for periodontal surgery. Before surgery, patients received cause-related periodontal therapy (SRP, oral hygiene instructions, plaque control). The surgical technique was a modified papilla flap for both groups, test and control. The authors used 0.2 mL of a gel containing 4 mg/mL rhFGF-2 in sodium hyaluronate MW 1.3 × 10^6^. After surgery, patients were informed to respect the oral hygiene protocol and were prescribed doxycycline as a prophylactic antibiotic.

Mamajiwala et al. [21] recruited patients that received phase I therapy (SRP, oral hygiene instructions). Then, 6 weeks after this therapy, infrabony defects with a PD interproximal ≥ 5 mm were considered for surgery. Test and control groups were treated using open-flap debridement with/without HA. The authors did not mention the dosages used for HA application. After surgery, patients were informed to respect the oral hygiene protocol and were prescribed ibuprofen.

### 3.4. Periodontal Parameter Assessment

**Figure 2:**
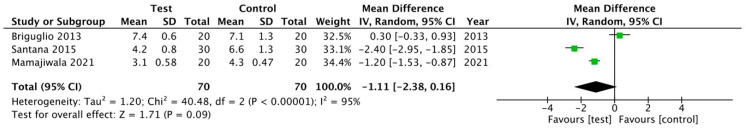
Forest plot for PD reduction after 12 months.

**Figure 3:**
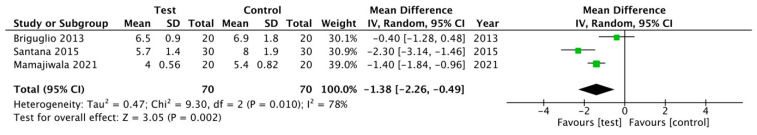
Forest plot for CAL gain after 12 months.

For the PD parameter (Figure 2), no statistical differences were found between the test group and control group; overall, the weighted mean (WM) was −1.11 mm (95% CI −2.38 to 0.16 mm; *p* = 0.09). The heterogeneity between RCTs was 95%. For the CAL parameter, (Figure 3), statistical significance was achieved favoring the treatment; the WM was −1.38 mm (95% CI −2.26 to −0.49 mm; *p* = 0.002). The heterogeneity between RCTs was 78%.

### 3.5. Risk of Bias Assessment

**Figure 4:**
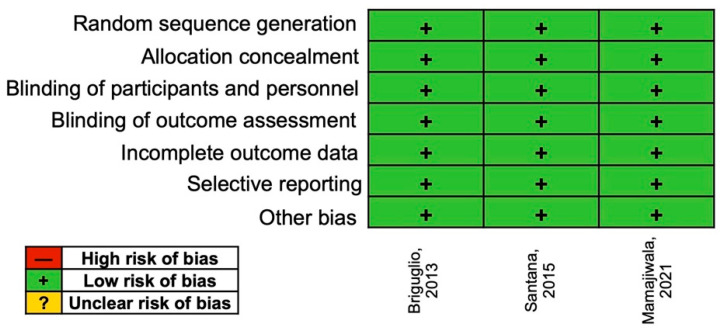
Risk of bias assessment.

According to the assessment using the Cochrane risk of bias, all three RCTs were considered at low risk (Figure 4).

## 4. Discussion

Over the years, surgical periodontal therapy has been performed to treat infrabony defects. Studies have demonstrated that osseous substitute materials, EMD, membranes, growth factors, and other possible variations of materials have been successfully used in the process of periodontal healing [11,22].

When it comes to the use of HA, this product has the capacity to absorb growth factors, due to its hygroscopic characteristics and antimicrobial and anti-inflammatory properties [22], with the ability to promote tissue healing. Furthermore, the presence of HA in the regulation of water equilibrium can be an advantage in the healing process, contributing to the cell hydration phenomenon [23]. Monheit and coworkers studied the properties of HA. The authors highlighted its binding affinity, a feature that confers HA the role of a physical support material, with specific roles in lubrication, shock buffering, or protein filtration [24]. These characteristics constitute essential requirements in the success of treating infrabony defects. An in vitro study conducted by Fujioka-Kobayashi et al. [25], investigating the impact of HA on periodontal ligament cell growth, development, and proliferation, demonstrated that crosslinked HA and non-crosslinked HA were both related to a substantial increase in periodontal cell count. Furthermore, the additional use of an osteogenic differentiation medium was able to produce a boost of alkaline phosphatase and collagen levels [25]. The advantages of HA in periodontal defects were also confirmed by Miranda et al., where HA was used in combination with chitosan, in order to develop an artificial structure with roles in periodontal ligament cell proliferation [26].

Another feature that needs to be taken into account is the ability of the HA matrix to adjust to the desired texture, by modulating the saline and blood content, promoting the insertion of the bone graft into the periodontal defect, due to its chemical and physical characteristics. Clinical and radiological outcomes from the Balilini study demonstrated the presence of bone growth and the absence of a major inflammatory reaction [12]. In the study of Bogaerde, following 19 cases of infrabony defects treated with esterified HA, it was concluded that the method stood out for its simplicity and fast applicability [13]. Furthermore, HA could induce CAL improvement and a decrease in deep PD. The authors underlined that the most successful outcomes were predicted for pathologies containing two or three wall lesions, while combined treatment approaches were indicated for more difficult cases [13].

In the study of Fawzy El-Sayed et al. [18], each patient initially received nonsurgical periodontal therapy consisting of scaling and root planning (SRP) plus oral hygiene instructions. Then, 8 weeks after SRP, a re-evaluation of the infrabony defects with PD interproximal > 5 mm was conducted, and those sites were considered eligible for surgery. The defects for the test group were treated using a modified Widman flap, with instrumentation of the root surface and bone defects using Gracey curettes; then, HA was applied to the defect. The authors indicated a significant improvement of the periodontal parameters (PD, CAL, PI) for the control and test groups.

In a meta-analysis performed by Eliezer and coworkers [7] assessing the use of HA in both nonsurgical and surgical therapy, it was concluded that HA may be able to offer additional benefits; however, the authors stated that a high risk of bias was assessed, and that more RCTs are needed to confirm the clinical success of this product. In this meta-analysis [7], only two surgical studies were included, where the WMs for CAL gain and PD were 0.85 mm and −0.89 mm, respectively, in favor of HA + OFD. Our results indicated WMs for CAL and PD of −1.38 mm and −1.11 mm, respectively, in favor of HA + OFD. These differences in results may be attributed to the inclusion of more RCTs.

An alternative to HA was described in the systematic review of Esposito et al. on EMD used in deep periodontal lesions [27]. This well-known product contains amelogenins, a group of proteins representative of the development of periodontal tissue and enamel. Although the use of amelogenins showed decreased postoperative clinical episodes, it was not demonstrated that it preserves more affected teeth or induces any aesthetic amelioration within 12 months of its usage [27]. On the other hand, Ramenzoti et al. proved that a combination of enamel matrix derivatives and HA leads to increased tissue regeneration and decreased inflammation [28]. A fusion of the two materials could, therefore, enhance the process of osseous healing in periodontal lesions [28]. A side-by-side comparison between amelogenins and HA was performed in the RCT of Pilloni and coworkers [11]. The authors of this paper concluded that the use of HA in a single-flap approach determined a PD reduction of 3.31 ± 0.70 mm compared to 4.5 ± 0.97 mm for amelogenins and a CAL gain of 2.19 ± 1.11 mm for HA compared to 2.94 ± 1.12 mm for amelogenins.

As can be seen in our review, the use of HA represents a relevant alternative in the treatment of infrabony defects. Among other therapeutic approaches that should be taken into account are alloplastic or natural polymers (chitosan, gelatin, and collagen) or ceramics (hydroxyapatite, tricalcium phosphate), which are widely known for their mechanical and biological properties that promote tissue regeneration [29]. This aspect was revealed in a systematic review, where polymer-based bone substitutes achieved greater filling of defects compared to OFD alone; however, they were inferior to bovine bone substitutes [30]. Another alternative that clinicians may find useful is the use of platelet-rich fibrin (PRF). PRF procedures performed in infrabony defects demonstrated the ability to induce a reduction in the periodontal lesions and an improvement of the clinical attachment compared to OFD alone [31,32].

Our review had several limitations. The first limitation was the low number of RCTs included, with a low number of patients and different periods of follow-up. A follow-up of 12 months was the most common time interval in the three RCTs included. The second aspect was the assessment of infrabony defects with different thresholds considered eligible for the surgery. The third aspect was the inability to complete a meta-analysis due to the heterogeneity of RCTs with different statistical analyses and a comparison of only PD and CAL parameters. In future RCTs, the use of CBCT pre- and postoperatively should be considered for an accurate assessment of the benefits of using HA in infrabony defects.

## 5. Conclusions

The use of hyaluronic acid in addition to periodontal surgery using open-flap debridement seems to have beneficial effects in terms of periodontal parameters (PD and CAL). To draw a clear conclusion, more adapted and well-designed clinical trials are needed to assess the advantage of this product in comparison with other products used in the treatment of infrabony defects.

## Figures and Tables

**Table 1:** Reasons for excluding articles.

Article	Reason for Exclusion
Pilloni [11]	RCT—comparison between EMD and HA with no control group
Ballini [12]	Prospective study with no control group
Bogaerde [13]	Prospective study with no control group
Bozic [14]	Prospective study—use of HA and deproteinized porcine bone with no control group
Bhowmik [15]	Prospective study with no control group
Engstrom [16]	Prospective study using guided tissue regeneration procedure
Sehdev [17]	Prospective study using guided tissue regeneration procedure
Fawzy El-Sayed [18]	RCT with follow-up of less than 12 months.

EMD: Emdogain; HA: hyaluronic acid; RCT: randomized clinical trial.

**Table 2:** Characteristics of the studies included.

Author, Year, Country	Study	Patients	Surgical Intervention	Results		Conclusions
Briguglio, 2013, Italy [19]	Parallel RCT	40 patients Male: 18 Female: 22 Mean age: 45 ± 8.2 years	Open-flap debridement	Baseline	Follow-up (12 months/24 months)	HA with OFD offered additional benefits in terms of PD reduction and CAL gain.
				PD: Control 8.0 ± 0.7 mm	PD: Control 7.1 ± 1.3 mm 7.2 ± 0.5 mm	
				Test 8.6 ± 1.1 mm	Test 7.4 ± 0.6 mm 7.0 ± 1.2 mm	
				CAL: Control 8.3 ± 1.2 mm	CAL: Control 6.9 ± 1.8 mm 7.2 ± 0.7 mm	
				Test: 7.2 ± 1.5 mm	Test: 6.5 ± 0.9 mm 5.3 ± 1.8 mm	
Santana, 2015, Brazil [20]	Split-mouth RCT	30 patients Male: 11 Female: 19 Mean age: 49.2 years Control group (*n* = 30 sites) Test group (*n* = 30 sites)	Open debridement with the papilla preservation flap	Baseline	Follow-up (1 year)	rhFGF-2/HA was able to improve periodontal parameters.
				PD: Control 9.5 ± 1.5 mm	PD: Control 6.6 ± 1.3 mm	
				Test 9.7 ± 1.9 mm	Test 4.2 ± 0.8 mm	
				CAL: Control 10.3 ± 1.3 mm	CAL: Control 8.0 ± 1.9 mm	
				Test: 10.4 ± 1.6 mm	Test: 5.7 ± 1.4 mm	
Mamajiwala, 2021, India [21]	Split-mouth RCT	20 patients Male: 11 Female: 9 Mean age: 39.9 ± 4.18 years Control group (*n* = 20 sites) Test group (*n* = 20 sites)	Open-flap debridement	Baseline	Follow-up (6 months/12 months)	HA in addition to OFD improved clinical and radiographic outcomes.
				PD: Control 8.45 ± 0.51 mm	PD: Control 5.2 ± 0.61 mm 4.3 ± 0.47 mm	
				Test 8.5 ± 0.94 mm	Test 4.5 ± 0.51 mm 3.1 ± 0.58 mm	
				CAL: Control 9.3 ± 0.73 mm	CAL: Control 6.8 ± 0.76 mm 5.4 ± 0.82 mm	
				Test: 9.15 ± 0.48 mm	Test: 5.6 ± 0.74 mm 4.0 ± 0.56 mm	

CAL: clinical attachment level; HA: hyaluronic acid; OFD: open-flap debridement; PD: probing depth; RCT: randomized clinical trial.

## Data Availability

Not applicable.
